# Antifungal Susceptibility and *Candida* sp. Biofilm Production in Clinical Isolates of HIV-Positive Brazilian Patients under HAART Therapy

**DOI:** 10.3390/biomedicines12020310

**Published:** 2024-01-29

**Authors:** Anelise Maria Costa Vasconcelos Alves, Érika Helena Salles de Brito, Márcio Flávio Moura de Araújo, Juliana Jales de Hollanda Celestino, Ana Caroline Rocha de Melo Leite, Gabriela Silva Cruz, Nuno Filipe Azevedo, Célia Fortuna Rodrigues

**Affiliations:** 1 Department of Morphology, Faculty of Medicine, Federal University of Ceará, Fortaleza 60430-170, Ceará, Brazil; anelise_alves@yahoo.com.br; 2 LEPABE—Laboratory for Process Engineering, Environment, Biotechnology and Energy, Faculty of Engineering, University of Porto, 4200-465 Porto, Portugal; nazevedo@fe.up.pt; 3 ALiCE—Associate Laboratory in Chemical Engineering, Faculty of Engineering, University of Porto, 4200-465 Porto, Portugal; 4 Institute of Health Sciences, University of International Integration of Afro-Brazilian Luso-Phony, Redenção 62790-000, Ceará, Brazil; erika@unilab.edu.br (É.H.S.d.B.); juliana.celestino@unilab.edu.br (J.J.d.H.C.); acarolmelo@unilab.edu.br (A.C.R.d.M.L.);; 5 FIOCRUZ—Oswaldo Cruz Foundation, Eusébio 61773-270, Ceará, Brazil; marcio.moura@fiocruz.br; 6 1H-TOXRUN—One Health Toxicology Research Unit, Cooperativa de Ensino Superior Politécnico e Universitário—CESPU, 4585-116 Gandra PRD, Portugal

**Keywords:** antifungals susceptibility, HIV, Candida species, oral candidiasis, biofilm, resistance

## Abstract

The aim of the present study was to characterize biofilms formed by *Candida* spp. clinical isolates (n = 19), isolated from the oral mucosa of HIV-positive patients. For characterizing the biofilms formed by several *Candida* sp. strains, isolated from HIV-positive patients, in terms of formed biomass, matrix composition and antifungal susceptibility profile, clinical isolates (n = 19) were collected from oral mucosa and identified. The biofilm of the samples was cultured with fluconazole (1250 mg/L), voriconazole (800 mg/L), anidulafungin (2 mg/L) or amphotericin B (2 mg/L). Afterwards, the quantification of the total biomass was performed using crystal violet assay, while the proteins and carbohydrates levels were quantified in the matrix. The results showed a predominance of *C. albicans*, followed by *C. krusei*. Around 58% of the *Candida* spp. biofilm had susceptibility to fluconazole and voriconazole (800 mg/L), 53% to anidulafungin and 74% to amphotericin B. *C. krusei* presented both the lowest and the highest biofilm matrix contents in polysaccharides and proteins. The low resistance to antifungal agents reported here was probably due to the fact that none of the participants had a prolonged exposure to these antifungals. A predominance of less virulent *Candida* spp. strains with low or no resistance to antifungals was observed. This can be attributed to a low fungal selective pressure. This most probably happened due to a low fungal selective pressure but also due to a good adherence to HAART therapy, which guarantees a stable and stronger immune patient response.

## 1. Introduction

Candidiasis is a common opportunistic infection in HIV-infected patients [1]. There has been a gradual emergence of *non-albicans Candida* (NCAC) species as a cause of refractory mucosal and invasive candidiasis, particularly in patients with advanced immuno-suppression, and the resistance to antifungal agents in the *Candida* species (particularly to azoles) is a point of concern [2,3,4]. Predisposing factors include age (children or older people), smoking, diabetes mellitus, nutritional disorders, endocrinopathies, immunosuppressive conditions (e.g., chemotherapy) and malignancies (e.g., cancer) [2,3,4]. 

In the past, oropharyngeal candidiasis (OC) was used as a way to diagnose AIDS by being one of the first clinical signs of this disease, affecting 50 to 95% of HIV-positive individuals [2,3,4,5,6]. OC can cause dysphagia, odynophagia, retrosternal chest pain and extreme weight loss [4]. The diagnosis is made on a clinical basis, based on endoscopic exam, which shows white mucosal plaque-like lesions, erythema and exudates adherent to the mucosa, or biopsy/mucosal brushing, in which is observed pseudohyphae of the *Candida* spp. Finally, microbiological confirmation, speciation and antifungal susceptibility are achieved through a culture of the endoscopically acquired samples [5]. In the 1970–80s, the treatment of OC was largely undertaken with fluconazole, and the enormous number of HIV-positive patients who have needed to be treated for OC has led to an increased *Candida* spp. resistance to azoles, particularly to fluconazole [3,7]. 

One of the most important mechanisms of antifungal resistance is the ability to form biofilm. Biofilms represent a type of microorganism community that adheres to biotic and abiotic surfaces; these microorganisms are inserted in an extracellular matrix that plays a key role in antimicrobial resistance and avoiding phagocytosis by immune system cells and provides physical barriers to environmental changes. In addition, the matrix plays a significant role in structure by forming water channels and promotes intercellular interaction [8,9]. The molecules that form the extracellular matrix are exopolysaccharides, nucleic acids (eDNA and eRNA), proteins, lipids (e.g., ergosterol) and other biomolecules [3,8]. Moreover, the biofilm matrix composition is directly associated with the pathogenicity of species/strains, as well as to the antifungal drug resistance [10]. *Candida* spp. that can form oral biofilms are more difficult to eradicate due to specific characteristics of this lifestyle form, resulting in chronic or recurrent oral infections [8].

A previous study has already shown that *C. albicans* strains isolated from HIV+ patients were weaker biofilm formers due to a lower adhesion capacity when compared with strains isolated from an HIV- group. In the same study, it was found that patients receiving highly active antiretroviral therapy (HAART) had a better response to antimicrobial treatments [11]. However, there are no data on the characterization of the biofilm matrix of *Candida* spp. strains isolated from HIV+ people. This would help to understand the pathogenesis of candidiasis, as well as the selection of the best treatment.

In this study, we investigated the prevalence of antifungal drug resistance in clinically isolated *Candida* from HIV-infected individuals to better understand the *Candida* spp. biofilm mechanisms and its matrix characterization.

## 2. Materials and Methods

### 2.1. Participants

All participants were diagnosed with HIV and had been on antiretroviral therapy (HAART) for at least 6 months. All patients were using nucleoside reverse transcriptase inhibitors and had no history of hospitalization due to HIV/AIDS complications. In addition, none of the patients were hospitalized or undergoing outpatient treatment for other diseases. They were of both sexes, aged ≥18 years and mentally capable of answering an interview. The exclusion criteria for the participants were HIV-associated neurocognitive disorder (score ≤11 on the International HIV Dementia Scale), and no medical/laboratory records of their TCD4 lymphocyte count for more than one year or record of illicit drug use in the previous 30 days.

Antiretroviral therapy was specific to each patient. The main drugs in use were nucleoside reverse transcriptase inhibitors (100%). The most common regimens consisted of lamivudine and tenofovir (49.5%), lamivudine and zidovudine (10.6%), lamivudine alone (8.7%) and zidovudine together with tenofovir (6.7%). A smaller proportion used non-nucleoside reverse transcriptase inhibitors [NNRTIs] (68%) and protease inhibitors (5.8%). The combination of atazanavir and ritonavir was the most widely used among protease inhibitors. Among NRTIs, efavirenz (75.7%) and nevirapine (24.3%) were the drugs of choice.

This study was conducted according to the guidelines of the Declaration of Helsinki and was approved by the Institutional Review Board from the Federal University for International Integration of the Afro–Brazilian Lusophony (UNILAB) under the number approval number: 2,691,682, following the ethical aspects of the resolution 466/12 and 510/16 of the National Health Council. After the presentation of the study and a review of all the criteria, the participants signed a copy of the informed consent form. All the names and private information of patients were kept confidential.

### 2.2. Microorganisms Isolation and Characterization of Microorganisms

Clinical isolates (swab from tongue or oral mucosa) of *Candida* spp. (n = 19) were collected by a lab technician from the oral mucosa of patients (n = 106), from a specialized out-patient clinic for the treatment of individuals with HIV/AIDS, located in Fortaleza, Brazil. Data collection was conducted between August and November of 2018. Afterwards, isolates were stored and kept at −80 °C, until accurate identification using biomolecular methods. The reference strains *Candida albicans* SC5314 and *Candida glabrata* ATCC2001 were acquired from the American Type Culture Collection (Manassas, VA, USA). In all cases, for routine identification, *Candida* isolates were grown in Sabouraud Dextrose Agar (SDA) (Merck, Darmstadt, Germany) under aerobic conditions for 24 h at 37 °C. The procedures for identification were performed by standard mycological methods at 30 °C for 48 h using chromogenic medium CHROMagar *Candida* (CHROMagar Microbiology, Paris, France) [9,11,12,13].

### 2.3. Inoculum Preparation

*Candida* spp. were grown on SDA and incubated for 24 h at 37 °C. To prepare the inoculum, cells were then inoculated in SDB Sabouraud dextrose broth (SDB) (Merck, Darmstadt, Germany) and incubated for 18 h at 37 °C under agitation at 120 rpm. After incubation, the inoculum density was adjusted to 1 × 10^5^ cells/mL using a Neubauer chamber with RPMI-1640 (Sigma-Aldrich, St. Louis, MO, USA) [8].

### 2.4. Antifungal Drugs

Fluconazole (Flu), voriconazole (Vcn) and anidulafungin (Afg) were provided by Pfizer^®^ (New York, NY, USA), in their pure form. Amphotericin B (AmB) came from Sigma^®^ (Sigma-Aldrich, Buffalo, NY, USA). Aliquots of 5000 mg/L of Flu, Vcn and 40 mg/L of AmB and Afg were prepared using dimethyl sulfoxide (DMSO) for all drugs. The final concentrations used were prepared in RPMI-1640.

### 2.5. Biofilm Growth and Characterization

The characterization of the *Candida* spp. strains’ biofilms was performed according to Alves et al. [9]. Briefly, a total of 100 μL of each strain inoculum was transferred to each well of the 96-well micro-plate and 100 μL of RPMI-1640, supplemented or not with antifungals (2× concentrated), was added, for 48 h at 37 °C. The antifungals tested were Flu (1250 mg/L) (New York, NY, USA)—Pfizer), Vcn (800 mg/L) (New York, NY, USA)—Pfizer), Afg (2 mg/L) (New York, NY, USA)—Pfizer) and AmB (2 mg/L) (Sigma-Aldrich, Buffalo, NY, USA). Wells containing only culture medium without inoculum were used as a negative control. After incubation, the biofilm biomass was analyzed using the crystal violet (CV) (Sigma-Aldrich, St. Louis, MO, USA) assay. For this, the supernatant was carefully aspirated, and the wells were washed twice with 200 μL PBS (Phosphate Buffered Saline, 0.1 M, pH = 7.2). Subsequently, biofilm was fixed by 100% (*v*/*v*) methanol, 200 μL/well, for 20 min. After drying, the supernatant was aspirated and 200 μL of 1% (*w*/*v*) aqueous CV was added to each well. After 5 min, the dye solution was aspirated, and the wells were washed twice with sterile distilled water. Subsequently, 200 μL of a 33% acetic acid solution was added to each well and immediately transferred to a new 96-well plate. Then, the plates were read at 570 nm (FLUOStar Omega Plate Reader, BMG LABTECH, Ortenberg, Germany) [8]. The cut-off optical density (ODc) was defined as three standard deviations above the mean OD of the negative control, and the strains were classified as follows: OD ≤ ODc = no biofilm producer; ODc < OD ≤ 2 × ODc = weak biofilm producer; 2 × ODc < OD ≤ 4 × ODc = moderate biofilm producer; and 4 × ODc < OD = strong biofilm producer [9,13].

### 2.6. Quantification of Matrix Polysaccharides

The quantification of polysaccharides was performed using phenol-sulfuric acid method. Briefly, the biofilm matrix was collected after incubation period, then the supernatant was sonicated, vortexed and centrifuged at 4000 rpm for 8 min. The supernatant was sterilized with a 0.22 μm filter membrane. Then, the filtrate (0.5 mL) was incubated with 0.5 mL of phenol (50 g/L) and 2.5 mL of sulfuric acid (95–97%) into glass tube at room temperature for 15 min. After, the plate was read for the absorbance at 490 nm using PBS as blank. The quantity of polysaccharides was extrapolated from a standard curve made with standard glucose concentrations. The quantity of polysaccharides should be normalized by weight of biofilm (mg polysaccharides/g biofilm) [9,13].

### 2.7. Quantification of Matrix Protein

The purified biofilm matrix (25 μL) was transferred to 96-well plate and added to a 200 μL of reagent mixture of BCA kit (Merck KGaA, Darmstadt, Germany) to each well. The BCA kit is a manufactured product suitable for measuring protein concentration by a reduction of copper (Cu2+) salt and colorimetric quantification. The solution was homogenized with pipette and incubated for 30 min at 37 °C. Then, the absorbance at 562 nm was determined using PBS as blank. The amount of protein was extrapolated from a standard curve performed with standard BSA concentrations. The amount of protein should be normalized by weight of biofilm (mg protein/g biofilm) [9,13].

### 2.8. Statistical Analysis

The experimental data were evaluated using GraphPad Prism v.9.1.1 software (San Diego, CA, USA). The data were analyzed using one-way ANOVA followed by Dunnett’s test. In all cases, statistical significance was set as *p* < 0.05. Data are presented as the mean ± standard deviation (SD). All experiments were performed three times independently, in triplicate. This study is experimental research, a quantitative survey.

## 3. Results

### 3.1. Identification and Characterization of Participants of Study

The sociodemographic characteristics of the participants are described in Table 1. Of the studied patients, a total of 40.6% were colonized by *Candida* spp. in the oral cavity; however, only 19 strains remained viable in the mycotheque and these were characterized in this study. No differences were observed between the study participants regarding the sociodemographic characteristics analyzed and the isolation of oral *Candida* spp. However, data on lifestyle showed a positive correlation between smoking and *Candida* isolation [14] (Table 1). In addition, more information about the patients can be accessed in the Supplementary Material, Tables S1 and S2.

### 3.2. Identification and Characterization of Biofilm Formation of Candida spp.

The results show that HIV positive patients presented a predominance of *C. albicans* (57%) (Table 2), followed by *C. krusei* (37%) as noted in the literature [15,16,17]. All *Candida* spp. isolates from HIV patients had the ability to form biofilms (Table 2). 

**Figure 1:**
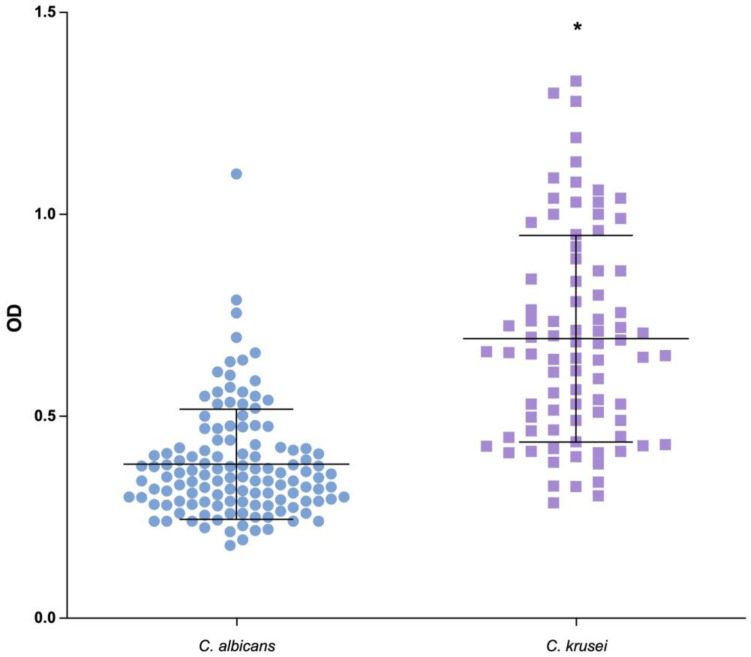
In vitro biofilm production by *C. albicans* and *C. Krusei* strains of clinical isolates from HIV patients. Biofilm was quantified through staining with CV, after 48 h of incubation. The clinical isolates were compared with the optical density of the reference strain (*C. albicans* SC5314). Each value is the average of three independent experiments in triplicate. Error bars are the standard deviations (* *p* < 0.0001).

Most of these strains were moderate biofilm formers (52%), followed by weak (37%) and strong (11%) biofilm formers. Curiously, none of the strongest biofilm formers’ strains were *C. albicans*—which normally produce strong biofilms [9,13]—but *C. krusei* H6 (biomass: 1.086 Abs/cm^2^ ± 0.41) (Figure 1). It is relevant to note that all these strains were isolated from oral microbiota and thus not necessarily with candidiasis.

### 3.3. Biofilm Matrix Composition

**Figure 2:**
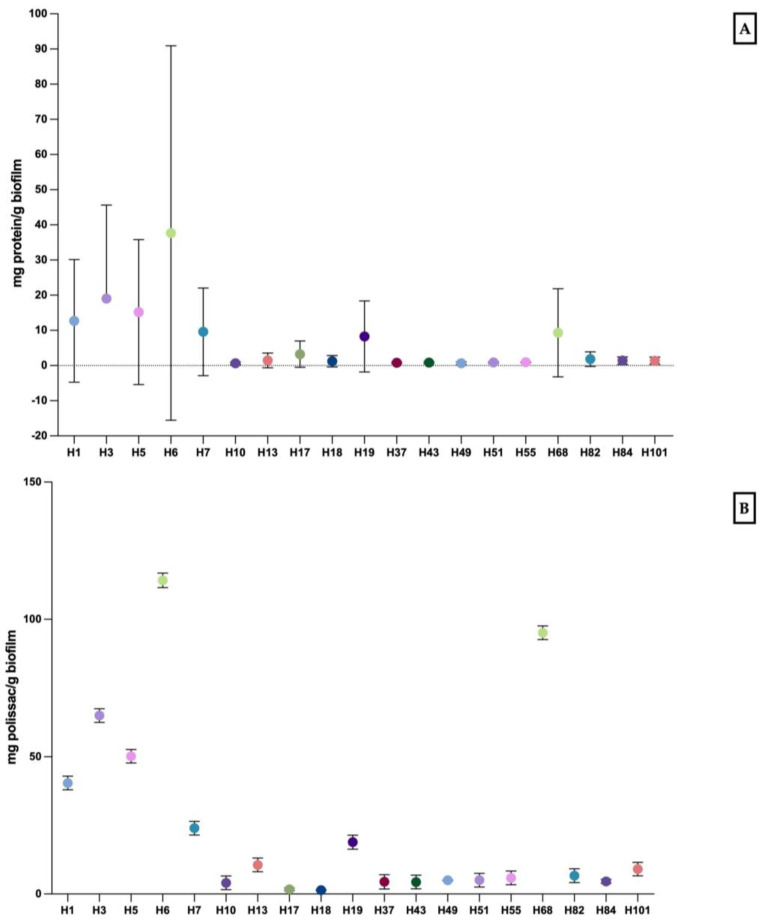
Matrix composition of *Candida* spp. biofilms isolated from HIV patients: mean values of protein quantity (mg/g of biofilm) (**A**) and mean values of polysaccharide quantity (mg/g of biofilm) (**B**) ± standard deviation (SD).

For the study of biofilm matrix composition, the polysaccharide and protein contents were determined (Figure 2). Interestingly, *C. krusei* presented as both the lowest and the highest contents in these two biomolecules. In terms of the dry weight of biofilm mg/L mg ± SD, it was observed that the lowest values were in H1 (0.054 ± 0.03) and the highest values in H6 (0.571 ± 0.56). The highest and lowest proteins’ contents were found in the biofilms of *C. krusei* H6 and *C. krusei* H10, respectively (Figure 2A). The lowest level of polysaccharides mg/g in a biofilm were found in the *C. krusei* H18 biofilm and the highest level of polysaccharide was observed in *C. krusei* H6 (Figure 2B).

### 3.4. Effect of Antifungals against Candida spp. Biofilms Formation

**Figure 3:**
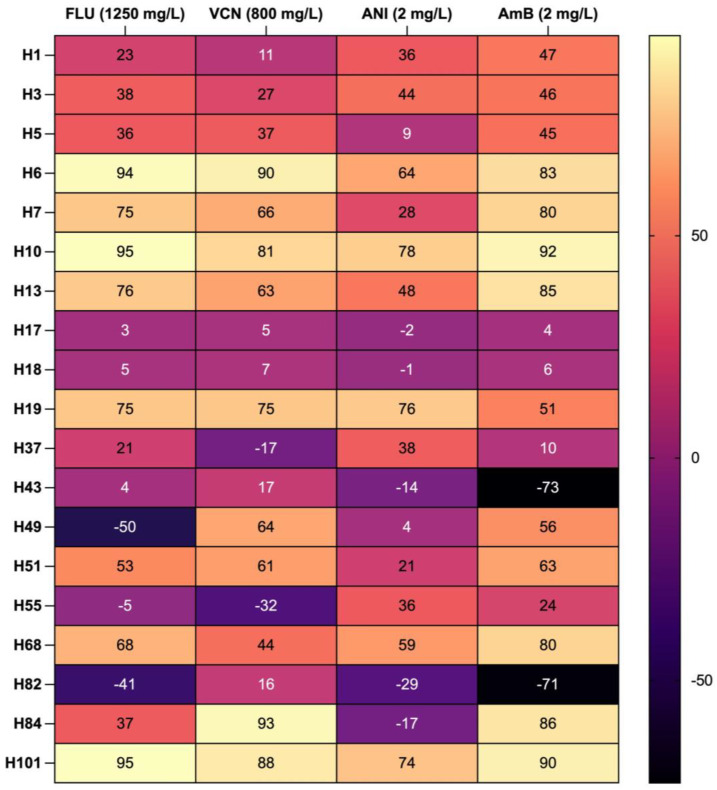
Heatmap of percentages reduction of biofilm formation in presence of antifungals.

Figure 3 presents the percentage of biomass reduction in the presence of Flu (1250 mg/L), Vcn (800 mg/L), Ani (2 mg/L) and AmB (2 mg/L). These concentrations were carefully chosen having been accounted in several previous studies of our group, in the same conditions (antifungal drugs to treat matured biofilms of *Candida* spp. [9,10,11,12,13]. Generally, the inhibition of the biofilm formation (prophylaxis) was achieved in the presence of antifungal drugs.

The highest biomass reduction was observed in strain H101 (*C. krusei*) in the presence of fluconazole (95%, *p* < 0.01). This strain was susceptible to all tested antifungal drugs. Although, at large, the strains from HIV-positive patients were susceptible to the antifungals, it is relevant to highlight strains *C. albicans* H17 and *C. krusei* H18, which were resistant to all antifungal drugs. Another point that draws attention are *C. albicans* isolates H5 and H7, which were susceptible to all antifungals, except for anidulafungin. *C. glabrata* H49 also demonstrated resistance to Ani. This is a clinically relevant result, since Ani belongs to the echinocandins’ class, a more recent class of antifungal drugs that are considered as first-line drugs for the treatment of systemic candidiasis [18]. Finally, other strains show a specific resistance to the azoles, for example, *C. albicans* H37 and H43.

## 4. Discussion

Highly active antiretroviral therapy (HAART) is a treatment used to control HIV infection using a combination of three or more antiretroviral drugs [19,20,21,22]. This combination therapy has primarily been indicated to treat human immunodeficiency virus type 1 (HIV-1)-infected patients. This combination could be among more than 28 different medications from six different classes [21]. Usually, the treatment starts with two nucleoside reverse transcriptase inhibitors plus one non-nucleoside reverse transcriptase inhibitor or integrase strand transfer inhibitor [22]. The aim of HAART is to reduce morbidity and mortality [23], improve immune function [24], reduce challenging opportunistic infections [25] and progressive multifocal leukoencephalopathy [26], reduce the viral RNA load in plasma [27], prevent drug resistance [28,29], reduce HIV transmission [30,31,32,33] and promote the well-being of HIV positive patients [34]. All these characteristics are related to the immune system and, consequently, to the pathogen–host relationship and the health–disease binomial. Thus, infections mainly caused by opportunistic pathogens have decreased in the post-HAART era. The relation between the prevalence of oral candidiasis and HAART-treated HIV-positive patients has been highlighted; once this disease was the most common oral opportunistic infection [1,17,35,36].

In this study, *C. albicans* was the most common species in HIV-infected patients with 57% of strains, followed by *C. krusei* (37%). *C. albicans* has been related by several studies as the most common [4,15,16,17], but among NCACs, the prevalence is controversial. Some studies have identified *C. glabrata* as the most common NCAC [4,16], on the other hand, other studies have identified *C. dubliniensis* [16] or *C. krusei* [17]. This result may be linked to intrinsic characteristics of the population studied. In the present study, all *C. krusei* strains were strong or moderate biofilm formers, which is concerning since biofilm formation is an important antifungal resistance mechanism [9]. In fact, it is acknowledged that a higher expression of virulence factors such as biofilm formation and the change in the epidemiology of NCACs may be related to the selection pressure exerted by the large-scale use of fluconazole in the treatment of candidiasis in HIV-positive patients in the past [15].

In our study, all participants diagnosed with HIV had been on HAART for at least 6 months, which may also explain the characteristics of the strains in this study. In the past, OC was recurrent in HIV-infected patients and the standard treatment was almost exclusively performed with azoles, e.g., fluconazole [3,7]. In the era of post-HAART, the incidence of OC has decreased significantly [16]. A translational study showed that the use of HAART promotes a shift to NCACs, which also correlates with an increase in the number of CD4+ T cells. As is well known, there is a relationship between OC caused by *C. albicans* and immunosuppression. Thus, it is hypothesized that patients treated for long periods with HAART, with CD4+ T cells < 200 cells/μL and reduced viral loads, will have an improved immune system and consequently a lower incidence of OC caused by *C. albicans* [16,35,36,37], and the emergence of NCACs colonizing the oral cavity of HIV-infected patients [16]. Our results corroborate these data in relation to having a predominance of less virulent strains and low or no resistance to antimicrobials, probably due to low selective pressure.

Regarding the biofilm matrix composition, both the polysaccharide and protein levels were highest in *C. krusei* H6, denoting a more virulent pattern of this strain. Normally, the polysaccharides in the extracellular matrix contribute to the antimicrobial resistance of *Candida* spp. biofilms [18,37]. For example, β-1,3 glucan, β-1,6 glucan and α-1,2-branched α-1,6 mannan, components for the extracellular matrix, form a complex that sequesters drugs as fluconazole and other azoles via non-covalent interactions [18]. In addition, a higher level of biomass and polysaccharides had been associated with genes to the delivery and production of the β-1,3 glucans (e.g., FKS1, FKS2, BGL2 and XOG1) [38,39,40]. They are also related to the matrix structure and the adherence of the biofilm cells to the surfaces and, consequently, to the drug resistance phenotype [12,41,42].

Most *Candida* spp. from HIV-positive patients presented an inhibition of biofilm formation in the presence of Flu (1250 mg/L), Vcn (800 mg/L), Ani (2 mg/L) and AmB (2 mg/L). These concentrations were previously used and have promoted the inhibition of *Candida* spp. biofilms [7]. Although some of the isolates are susceptible to certain antifungal agents based on antifungal susceptibility testing, there were strains that were more resistant than others and had variation in susceptibility, depending on the drug tested, on biofilm formation. This could be explained by the fact that biofilm formation is complex and depends on a series of characteristics of the microorganisms and their interactions with the environment [8].

Even if *C. krusei* is usually considered inherently resistant to azoles [3], in our experiments, *C. krusei* H101 was susceptible to all tested antifungal drugs, and the highest biomass reduction was observed in this strain in the presence of Flu (95%, *p* < 0.01). This is a positive result, as this strain is a moderate biofilm former. In contrast, H17 (*C. albicans*) and H18 (*C. krusei*) strains were resistant to all antifungal drugs and were also moderate biofilm formers. In this study, the data collected on the matrix composition cannot explain the more aggressive behavior, i.e., virulence, since susceptible strains had rich biofilm matrices in proteins and/or polysaccharides. Naturally, this confirms that the pathogenic mechanisms of *Candida* spp. are both associated with host conditions and *Candida* spp. virulence factors [38,39,40,41,42,43,44], such as the biofilm formation [3], the overactivity of efflux pumps [44], altered sterol synthesis [43] and quorum sensing [39,43]. Another virulence factor associated with biofilm formation is the expression of resistance genes [40]. It is known that biofilms of *C. albicans* present a higher transcription of MDR1 and CDR1 than in planktonic cultures of the same age [44].

*C. albicans* H5 and *C. albicans* H7 showed as being less susceptible to Ani, which acts by inhibiting (1 → 3)-β-D-glucan synthase, an important enzyme in fungal cell wall synthesis. This is a significant clinical result, because, since 2016, echinocandins are first-line antifungal drugs used to treat candidiasis [18]. Acquired or intrinsic FKS1 point mutations in *C. albicans*, *C. tropicalis*, *C. glabrata* and *C. krusei* have been linked to these echinocandin resistance cases [38]. Goulart et al. [14] have shown that samples from HIV-positive patients that had received HAART presented *Candida* spp. isolates with 84% sensitivity, 15% DDS and 1% resistance to Flu; 99% sensitivity and 1% resistance to ketoconazole; and 73% sensitivity, 23% DDS and 4% resistance to itraconazole. Our findings show a higher rate of resistance than Goulart et al. [4] (only 58% sensitivity to Flu and Vcn (800 mg/L), 53% sensitivity to Ani and 74% sensitivity to AmB). These results may be explained because this author has investigated the antifungal susceptibility of fungi in planktonic form, which is a more susceptible stage to antimicrobial than biofilm [4], and because of the sample size of this study. Recently, a study exploring biofilm-producing *Candida* spp. causing OC infections among HIV patients in Nepal showed that, while *Candida* isolates in planktonic stages were susceptible to antifungals (fluconazole—25 μg, ketoconazole—15 μg, clotrimazole—10 μg and amphotericin B—10 μg), in biofilm form, they showed a high resistance (51.9%) to ketoconazole [45].

In our study, there were no reports of the use of recreational drugs by patients on HAART, except for alcohol. However, it is worth noting that the use of recreational drugs such as amphetamines, hallucinogens, opiates or alcohol can lead to the development of drug–drug interactions (DDIs). In particular, non-nucleoside reverse transcriptase inhibitors, protease inhibitors, integrase inhibitors, chemokine receptor 5 antagonists and HIV fusion inhibitors have several DDIs related to the interaction with cytochrome P450 or P-glycoprotein, which would interfere with the patient’s treatment and immune function [46].

In HIV-infected patients, the development of HAART has caused a change in the opportunistic infection patterns, as in OC. In this study, we observed no resistance to conventional antifungal therapy, which may have occurred due to a reduction in the number of clinical candidiasis cases and, thus, the decrease in the antifungal drugs’ use. Nevertheless, further epidemiological studies are needed to understand the global reality of the OC and drug resistance in HIV-infected patients.

## Figures and Tables

**Table 1:** Sociodemographic characteristics of the participants (according to Silva et al. [14]).

Variables	N	%
Gender		
Male	68	64.2
Female	38	35.8
Skin Color		
White	21	19.8
Black	29	23.4
Others	56	52.8
Smoking		
Yes	33	31.2
No	73	68.8
Alcohol consumption		
Never	63	19.8
Once a month	22	23.4
2–3 times a month	16	52.8
2–3 times a week	3	34
>4 times a week	1	40.6
Sexual orientation		
Heterosexual	54	50.9
Homosexual	41	38.7
Bisexual	11	10.4

**Table 2:** Identification and data associated with biofilm production to each strain from HIV patients.

Strain Code	Species	Biofilm
H1	*Candida albicans*	Weak
H3	*Candida albicans*	Weak
H5	*Candida albicans*	Moderate
H6	*Candida krusei*	Strong
H7	*Candida albicans*	Weak
H10	*Candida krusei*	Strong
H13	*Candida krusei*	Moderate
H17	*Candida albicans*	Moderate
H18	*Candida krusei*	Moderate
H19	*Candida krusei*	Moderate
H37	*Candida albicans*	Moderate
H43	*Candida albicans*	Moderate
H49	*Candida glabrata*	Weak
H51	*Candida albicans*	Moderate
H55	*Candida albicans*	Weak
H68	*Candida albicans*	Weak
H82	*Candida albicans*	Weak
H84	*Candida krusei*	Moderate
H101	*Candida krusei*	Moderate

## Data Availability

The data that support the findings of this study are available on request from the corresponding author.

## Supplementary Materials

The following supporting information can be downloaded at: https://www.mdpi.com/article/10.3390/biomedicines12020310/s1, Table S1: Characteristics and lifestyle habits of the participants according to Silva et al. [14]); Table S2: Clinical characteristics of the participants according to Silva et al. [14]); Table S3. Identification and Characterization of Biofilm Formation of *Candida* sp.; Figure S1: In vitro biofilm production of clinical isolates from HIV patients. Biofilm was quantified through staining with CV, after 48 h of incubation.
