# Supplementary material for: Antifungal Susceptibility and Candida sp. Biofilm Production in Clinical Isolates of HIV-Positive Brazilian Patients under HAART Therapy
Source: Biomedicines. 2024 Jan 29;12(2):310. doi: 10.3390/biomedicines12020310 (PMC10886575; doi:10.3390/biomedicines12020310)
Supplement: Supplementary file 1 [file biomedicines-12-00310-s001.zip › biomedicines-2791857-supplementary.pdf]

Table S1. Characteristics and lifestyle habits of the participants according to Silva et al. [14].

Variables	N	%
Physical exercise		
Yes	37	35
No	69	65
Relationship status		
Married	36	34
Single	43	40.6
Widower	03	2.8
Divorced	10	9.4
Partner	14	13.2
Employment status		
Employed	36	34
Unemployed	03	2.8
Student	10	9.4
Others	57	53.8
Social class		
Lower (<$1500)	77	72.6
Middle ($1500–4500)	26	24.5
Upper (>$4500)	03	2.8

Table S2. Clinical characteristics of the participants according to Silva et al. [14].

Variables	N	%
TCD4 counts		
≥200 cells/mm^3	83	19.8
<200 cells/mm^3	7	23.4
TCD8 counts		
≥200 cells/mm^3	65	35
<200 cells/mm^3	0	65

Table S3. Identification and Characterization of Biofilm Formation of *Candida* sp.

Strain Code	Specie	O.D.570	Biofilm	Sample
H1	*Candida albicans*	0.315 ± 0.06	Weak	Oral swab
H3	*Candida albicans*	0.329 ± 0.04	Weak	Oral swab
H5	*Candida albicans*	0.579 ± 0.16	Moderate	Oral swab
H6	*Candida krusei*	1.086 ± 0.16	Strong	Oral swab
H7	*Candida albicans*	0.27 ± 0.03	Weak	Oral swab
H10	*Candida krusei*	0.967 ± 0.09	Strong	Oral swab
H13	*Candida krusei*	0.577 ± 0.16	Moderate	Oral swab
H17	*Candida albicans*	0.577 ± 0.13	Moderate	Oral swab
H18	*Candida krusei*	0.458 ± 0.07	Moderate	Oral swab
H19	*Candida krusei*	0.485 ± 0.63	Moderate	Oral swab
H37	*Candida albicans*	0.410 ± 0.62	Moderate	Oral swab
H43	*Candida albicans*	0.387 ± 0.54	Moderate	Oral swab
H49	*Candida glabrata*	0.277 ± 0.03	Weak	Oral swab
H51	*Candida albicans*	0.303 ± 0.54	Moderate	Oral swab
H55	*Candida albicans*	0.348 ± 0.11	Weak	Oral swab
H68	*Candida albicans*	0.330 ± 0.04	Weak	Oral swab
H82	*Candida albicans*	0.314 ±0.05	Weak	Oral swab
H84	*Candida krusei*	0.686 ± 0.06	Moderate	Oral swab
H101	*Candida krusei*	0.687 ± 0.06	Moderate	Oral swab

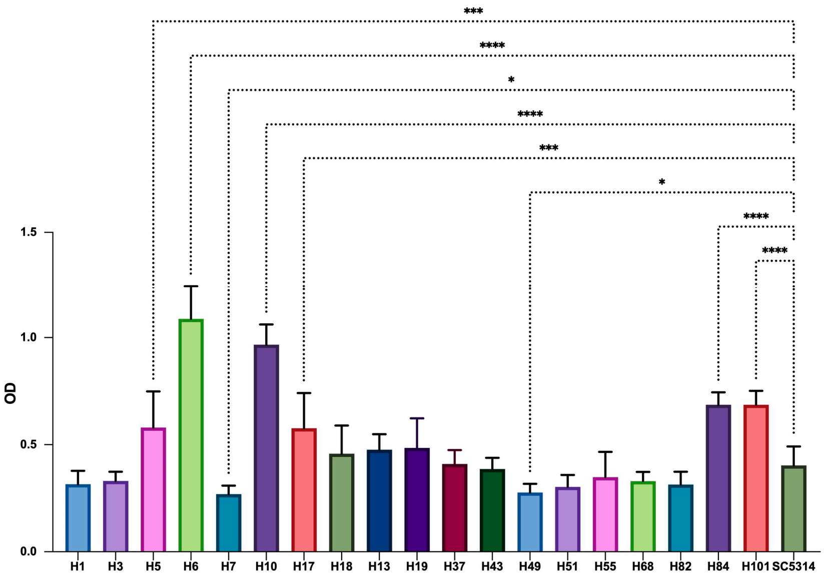

Figure S1. In vitro biofilm production of clinical isolates from HIV patients. Biofilm was quantified through staining with CV, after 48 h of incubation. The clinical isolates were compared with the optical density of the reference strain (*C. albicans* SC5314). Each value is the average of three independent experiments in triplicate. Error bars are the standard deviations (**** $p < 0.0001$; *** $p < 0.001$; * $p \geq 0.05$).
